# Early Detection of Age‐Related Decline of Muscle Cell Mass by Intracellular Water Assessment Compared With MRI or DXA

**DOI:** 10.1002/jcsm.13851

**Published:** 2025-06-04

**Authors:** Akifumi Maeda, Yosuke Yamada, Maito Yamagishi, Yoko Okada, Tsukasa Yoshida, Yuta Otsuka, Takayuki Izumo, Tomohiro Rogi, Hiroshi Shibata, Masahiro Fukuda, Takuma Arimitsu, Naokazu Miyamoto, Takeshi Hashimoto

**Affiliations:** ^1^ Faculty of Sport and Health Science Ritsumeikan University Kusatsu Shiga Japan; ^2^ Development & Design Department Suntory Beverage & Food Ltd. Kawasaki Kanagawa Japan; ^3^ National Institutes of Biomedical Innovation, Health and Nutrition Settsu Osaka Japan; ^4^ Department of Sports and Health Sciences, Graduate School of Biomedical Engineering Tohoku University Sendai Miyagi Japan; ^5^ Department of Medicine and Science in Sports and Exercise, Graduate School of Medicine Tohoku University Sendai Miyagi Japan; ^6^ Institute for Science of Life, Suntory Wellness Ltd. Seika Kyoto Japan; ^7^ Research Strategy Planning Department Suntory Holdings Ltd. Seika Kyoto Japan; ^8^ Fukuda Clinic Osaka Osaka Japan; ^9^ Undergraduate Department of Human Health, Faculty of Health Care Hachinohe Gakuin University Hachinohe Aomori Japan; ^10^ Graduate School of Health and Sports Science Juntendo University Inzai Chiba Japan

**Keywords:** ageing, bioelectrical impedance spectroscopy (S‐BIS), dual‐energy x‐ray absorptiometry (DXA), magnetic resonance imaging (MRI), muscle atrophy, muscle cell mass, sarcopenia, skeletal muscle mass

## Abstract

**Background:**

Ageing is commonly associated with a decrease in muscle strength, which is largely linked to decreases in skeletal muscle mass (SMM). The rate of the age‐related decline in muscle strength is different from the rate of the age‐related decline in SMM. Current estimation methods for SMM, such as magnetic resonance imaging (MRI) and dual‐energy x‐ray absorptiometry (DXA), evaluate both intracellular and extracellular water volumes, potentially overestimating atrophied muscle fibres. Recently, segmental bioelectrical impedance spectroscopy (S‐BIS), which distinguishes intracellular water (ICW) associated with muscle cell mass (MCM), has been developed. However, this method has not been sufficiently compared with other SMM assessment methods, especially with respect to age‐related changes. This study was aimed at comparing the age‐related differences in SMM measured by S‐BIS, MRI and DXA over a wide range of ages and to identify the optimal method for evaluating age‐related muscle tissue changes.

**Methods:**

This cross‐sectional study included 41 women and 43 men living in Japan (mean [SD] age: 53.51 [13.76] years; height: 162.96 [9.16] cm; BMI: 22.32 [2.94]). Three different methods were applied to estimate SMM: leg lean mass was measured via DXA; mid‐thigh muscle cross‐sectional area (CSA) was measured via MRI; and thigh total water, thigh ICW and the ratio of extracellular water to total water (ECW/TW) were measured via S‐BIS. To more effectively compare age‐related differences in each parameter, we normalised the relative values for each participant by using the mean scores for the 30–39‐year group for each parameter. Segmented regression analysis was performed to detect age breaking points for each parameter.

**Results:**

Each SMM parameter obtained via the different methods markedly decreased with increasing age (*p* < 0.05). In contrast, the ECW/TW increased with increasing age (*p* < 0.05). The age breaking points for leg lean mass measured by DXA, mid‐thigh CSA measured by MRI and thigh ICW measured by S‐BIS were 57.7, 56.3 and 43.2 years, respectively (*p* < 0.05).

**Conclusions:**

Compared with DXA or MRI, S‐BIS was found to be more sensitive for detecting age‐related SMM decrease at earlier stages. S‐BIS can distinguish between ICW and ECW, providing a more accurate estimation of MCM that is linked to muscle contractions. This study offers new insights that SMM begins to decrease in the 50s, while MCM does in the early 40s. These findings emphasise the need to address the age‐related decline in MCM starting in the early 40s to prevent a decrease in the quality of life of older individuals.

## 1 Introduction

Sarcopenia is characterised by the progressive loss of skeletal muscle mass (SMM) and strength, which affects physical function among older adults [1, 2, 3]. Sarcopenia increases the risk of frailty, institutionalisation, mobility disability and mortality. Thus, early detection of symptoms of sarcopenia and sustained application of management strategies are crucial.

There are various causes for age‐related decline in muscle strength, such as neurological changes, the hormonal and metabolic milieu, proinflammatory cytokines and fat infiltration; furthermore, a decrease in SMM is considered to be a major risk factor [3, 4]. However, the rate of the age‐related decline in muscle strength is different from the rate of the age‐related decline in SMM, and previous studies suggest that the age‐related decline in muscle strength begins before the age‐related decrease in SMM [4, 5, 6]. Skeletal muscle tissue is composed of myofibers, which are formed from muscle cells, and surround intermuscular adipose tissue (IMAT), connective tissue and extracellular water (ECW) [7, 8]. Since skeletal muscle contractions are caused by myofibers, we consider muscle cell mass (MCM) to be a more important index than SMM.

Muscle tissue contains nearly 70% water, which is distributed intracellularly and extracellularly; furthermore, it is believed that the volume of intracellular water (ICW) reflects MCM in the limbs [9, 10]. The most commonly used methods for estimating regional muscle mass are the muscle cross‐sectional area (CSA) via magnetic resonance imaging (MRI) or lean mass via dual‐energy x‐ray absorptiometry (DXA). However, owing to the measurement principles of these methods, they cannot differentiate between ICW and ECW volumes [11, 12]. Muscle fibre atrophy with ageing causes changes in the ratio of ICW to ECW in skeletal muscle, resulting in a higher ECW/ICW ratio in older individuals than in young adults [13]. Therefore, MRI and DXA may underestimate actual muscle cell atrophy during ageing [9].

Recently, segmental bioelectrical impedance spectroscopy (S‐BIS) has been developed to measure segmental ICW and ECW separately. The most significant difference between the S‐BIS and the conventional single‐frequency bioelectrical impedance analysis (BIA) methods is that S‐BIS can estimate the ICW and ECW separately by using multiple electrical frequencies. It has been reported that the ICW volume reflects MCM, which is closely associated with muscle contraction [13]. In fact, previous studies have reported that the ICW estimated by S‐BIS is strongly associated with age‐related declines in muscle strength and function [9, 14]. Therefore, S‐BIS is considered to be a more sensitive method than MRI or DXA for detecting age‐related muscle ‘cell’ atrophy. However, there is no direct comparison between the age‐related changes in muscle CSA estimated by MRI, lean mass estimated by DXA and ICW estimated by S‐BIS. This study was aimed at comparing age‐related muscle atrophy in muscle mass measured by S‐BIS, MRI and DXA in women and men over a wide range of ages and identifying the optimal method for evaluating age‐related muscle tissue changes.

## 2 Methods

### 2.1 Study Design

This cross‐sectional study included community‐dwelling individuals living in Japan. The subjects were recruited for the study based on the following inclusion and exclusion criteria. The inclusion criteria were as follows: women and men aged 30–79 years at the time of informed consent; those who had not exercised regularly (i.e., at least twice a week for at least 30 min per session over 1 year prior to the study), those who could visit the designated study site on the scheduled visit date, those who fully understood the purpose of the study and its contents and those who provided written consent. The exclusion criteria were as follows: the presence of locomotor disorders; a history of bone, joint or muscle‐related diseases such as fracture, sprain or separation within 3 months prior to the study; the presence or history of cardiovascular diseases; the presence of heart disease, liver disease, kidney disease, respiratory disease, diabetes mellitus, a positive for infectious diseases or other serious diseases; planning weight loss; having experience of high‐intensity exercise, such as bodybuilding or full marathon running; not eligible for MRI measurements due to having magnetic materials, tattoos or claustrophobia; an irregular lifestyle, such as shift workers and late‐night workers; a high level of alcohol consumption (more than 60 g/day); a high level of smoking (more than 21 cigarettes/day); a consumption of drugs or supplements, such as antihypertensive agents, protein, amino acids or vitamin D that may affect body water content and muscle mass; using a cane, supporter and so on on a daily basis; a systolic blood pressure of 160 mmHg or higher or a diastolic blood pressure of 100 mmHg or higher; pregnant (including possible pregnancy) or lactating; current participation in another study or within 4 weeks of the completion of another study; and the presence of any medical condition that was judged by the medical investigator to warrant exclusion.

By setting the effect size at 0.4, the alpha error at 0.05 and the power at 80%, we calculated that the number of subjects needed to perform a one‐way analysis of variance (ANOVA) in five groups (30–39‐, 40–49‐, 50–59‐, 60–69‐ and 70–79‐year age) was 80. Therefore, we recruited more than 80 subjects. This study was conducted at the Biwako Kusatsu Campus of Ritsumeikan University and at the Esaka Research Center of Soiken Inc. from April 22, 2018, to June 30, 2018. All the measurements and analyses were conducted by researchers with several years of experience. Additionally, the DXA measurements were performed by a certified radiologic technologist. All participants provided written informed consent before participating. The study protocol received approval from the Ethics Committee of Fukuda Clinic Research (Approval No. IRB‐20180317‐7) and Ritsumeikan University (Approval No. BKC‐2017‐084) and adhered to the principles outlined in the Declaration of Helsinki.

### 2.2 S‐BIS Measurements

**FIGURE 1:**
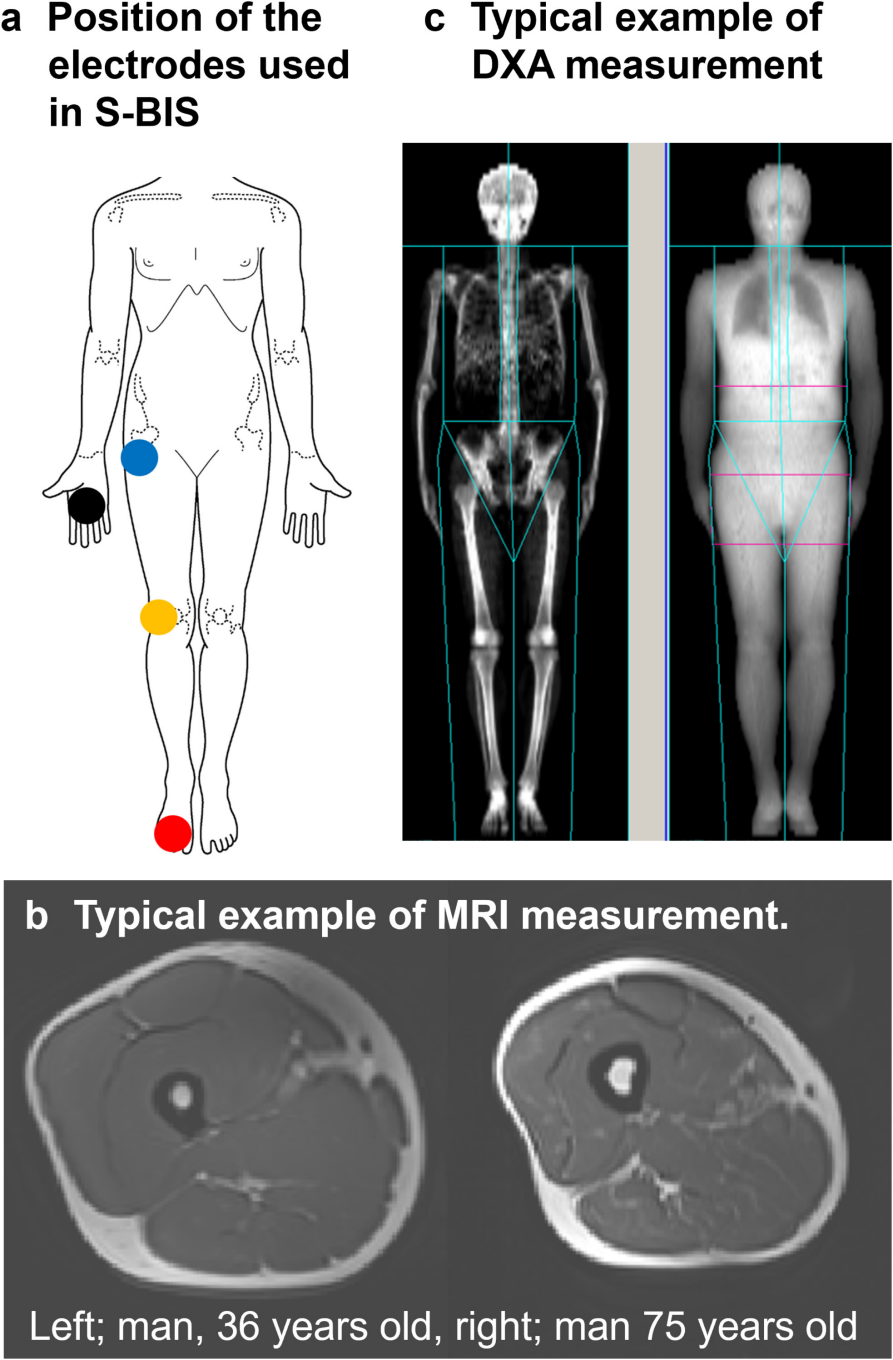
Explanation of each measurement of skeletal muscle mass. (a) Position of the electrodes used in segmental bioelectrical impedance spectroscopy (S‐BIS) measurement. The black and red points represent the injection electrodes. The blue and yellow points represent the sensing electrodes. (b) Typical example of the cross‐sectional area (CSA) via magnetic resonance imaging (MRI). Left: man, 36 years old; right: man, 75 years old. (c) Typical example of dual‐energy x‐ray absorptiometry (DXA) measurement. The light blue and pink lines indicate the boundaries of each site.

In accordance with previously described methods [9, 13, 15, 16, 17, 18], bioelectrical impedance was assessed via an SFB7 device (ImpediMed, Pinkenba, QLD, Australia) with disposable tab‐type monitoring electrodes (2 × 2 cm, Red Dot, 3M, St. Paul, MN, United States). An injecting electrode was placed on the dorsal surface of the right foot, proximal to the second and third metatarsophalangeal joints. Another injecting electrode was placed on the dorsal surface of the right hand, proximal to the second and third metacarpophalangeal joints. A sensing electrode was positioned on the right side of the body at the articular cleft between the femoral and tibial condyles and the anterior superior iliac spine (Figure 1a). The segment length (*L*) was calculated from the anterior superior iliac spine of the femur to the lateral tibial malleolus. Resistances at zero (*R*0) and infinite (*R*∞) frequencies were determined via extrapolation after the spectrum of bioimpedance data was fit to the Cole–Cole model via Bioimp software (ImpediMed), and *Ri* was calculated as 1/[(1/*R*∞) − (1/*R*0)]. The ECW was calculated as 99 × *L* ^2^/*R*0 for women and 98 × *L* ^2^/*R*0 for men, and the ICW was calculated as 281 × *L* ^2^/*Ri* for both women and men [18]. The total water content was calculated as ICW + ECW. To verify the reliability and validity of the measurements and analyses, the intraclass correlation coefficients (ICCs [1]) were calculated using values from two measurements conducted on a subset of participants (*N* = 16) with a 12‐week interval between the measurements. The ICC (1) of the ICW and ECW were 0.94 and 0.96, respectively.

### 2.3 MRI Measurements

T1‐weighted cross‐sectional MRI images were obtained for each leg from the superior border of the patella to the greater trochanter, including the rectus femoris muscle via a body array and spine coils (Body 18 and CP Spine Array Coil, Siemens Healthineers, Erlangen, Germany) with the following basic parameters: field of view, 420 × 420 mm; slice thickness, 10 mm; gap, 0 mm; pixel size, 1.75 × 1.31 mm; matrix size, 320 × 240; TR, 500 ms; TE, 11 ms; flip angle, 120°; band width, 435 Hz/pixel; and number of slices, 23 × 2 blocks. The participants lay supine with their legs extended and their muscles relaxed in a 3‐T magnet bore (MAGNETOM Skyra, Siemens Healthineers, Germany), as described previously (Figure 1b) [17]. From the 10‐mm‐thick slices, the images of the right mid‐thigh at the centre of the end‐to‐end images were analysed to measure the thigh CSA via SliceOmatic Ver 4.3 software (TomoVision, Magog, Canada). IMAT was manually excluded from the analysis when the thigh CSA was calculated according to the procedure in the previous study [19]. To verify the reliability and validity of the measurements and analyses, the ICC (1) was calculated using values from two measurements conducted on a subset of participants (*N* = 17) with a 12‐week interval between the measurements. The ICC (1) of thigh CSA was 0.98.

### 2.4 DXA Measurements

A DXA (Lunar iDXA; GE Healthcare United Kingdom Limited, Buckinghamshire, United Kingdom) was used for whole‐body composition assessment as described previously [17]. All the measurements were performed by a medical technologist at Ritsumeikan University. Lean mass was obtained from the whole body and leg regions (Figure 1c). Lean mass represents the sum of total body water, soft tissue mineral mass, protein and so on [12]. To verify the reliability and validity of the measurements and analyses, the ICC (1) was calculated using values from two measurements conducted on a subset of participants (*N* = 17) with a 12‐week interval between the measurements. The ICC (1) of lean mass was 0.98.

### 2.5 Statistical Analysis

G*Power 3.1.9.7 was used to calculate the sample size. The ICC was calculated manually via Microsoft Excel (2411). To compare age‐related differences in each parameter more effectively, we normalised the relative values for each participant by using the mean scores for the 30–39‐year groups of women and men for each parameter as a baseline of 100. One‐way ANOVA for the 30–39‐, 40–49‐, 50–59‐, 60–69‐ and 70–79‐year age groups was performed by using JMP (18.0.1). We performed receiver operating characteristic (ROC) analysis for ICW measured by S‐BIS, lower limb lean mass measured by DXA and thigh CSA measured by MRI via IBM SPSS (28.0.0) by setting the cutoff point at 44 years of age, as indicated by previous studies on age‐related changes [20]. We then performed segmented regression analysis to identify the age at which the change in each parameter occurred, that is, to detect an age breakpoint for each parameter, via SegRegA software (www.waterlog.info, accessed on 21 July 2024). The significance level was set at *α* = 0.05.

## 3 Results

Forty‐one female and 43 male adults aged from 30 to 77 years participated in this study. At least six women and seven men were included in each of the following age groups: 30–39, 40–49, 50–59, 60–69 and 70–79 years (Table 1).

**TABLE 1:** Characteristics of the participants. The values are expressed as the means ± standard deviations.

	Age group (years)	30–39	40–49	50–59	60–69	70–79
Women	*N*	9	9	9	6	8
	Age (years)	34.78 ± 3.01	45.11 ± 2.23	54.67 ± 2.49	64.83 ± 3.13	71.63 ± 2.29
	Height (cm)	158.71 ± 4.69	155.88 ± 4.07	155.41 ± 4.58	154.62 ± 2.88	152.60 ± 2.15
	Weight (kg)	51.22 ± 7.24	51.73 ± 4.23	53.80 ± 6.16	46.27 ± 2.85	50.81 ± 5.92
	BMI	20.30 ± 2.47	21.31 ± 1.85	22.30 ± 2.60	19.34 ± 0.82	21.77 ± 2.00
Men	*N*	9	9	9	9	7
	Age (years)	34.44 ± 2.79	43.89 ± 2.88	53.44 ± 2.71	65.56 ± 1.64	73.14 ± 2.53
	Height (cm)	175.40 ± 4.70	171.57 ± 4.36	169.97 ± 4.85	169.49 ± 6.28	165.31 ± 3.72
	Weight (kg)	68.10 ± 5.84	70.32 ± 9.00	69.66 ± 7.05	69.67 ± 9.57	63.51 ± 10.50
	BMI	22.19 ± 2.33	23.83 ± 2.29	24.09 ± 2.02	24.37 ± 4.04	23.12 ± 2.96

**FIGURE 2:**
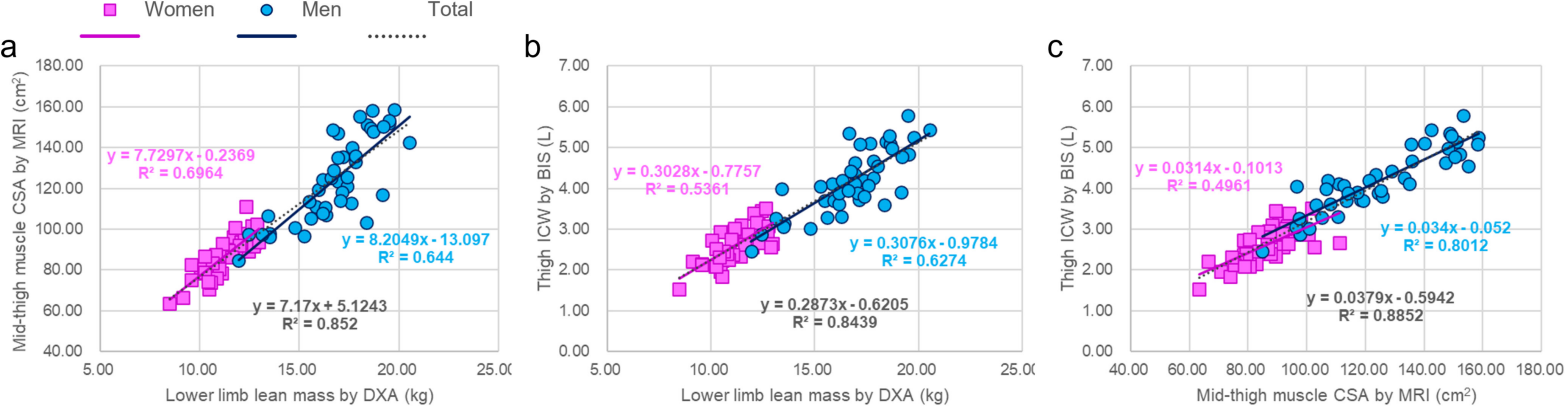
The correlation of the absolute values of each measurement. (a) Lower limb lean mass via dual‐energy x‐ray absorptiometry (DXA) vs. the cross‐sectional area (CSA) via magnetic resonance imaging (MRI). (b) Lower limb lean mass via dual‐energy x‐ray absorptiometry (DXA) vs. thigh intracellular water (ICW) via segmental bioelectrical impedance spectroscopy (S‐BIS). (c) The cross‐sectional area (CSA) via magnetic resonance imaging (MRI) vs. thigh intracellular water (ICW) via segmental bioelectrical impedance spectroscopy (S‐BIS).

**FIGURE 3:**
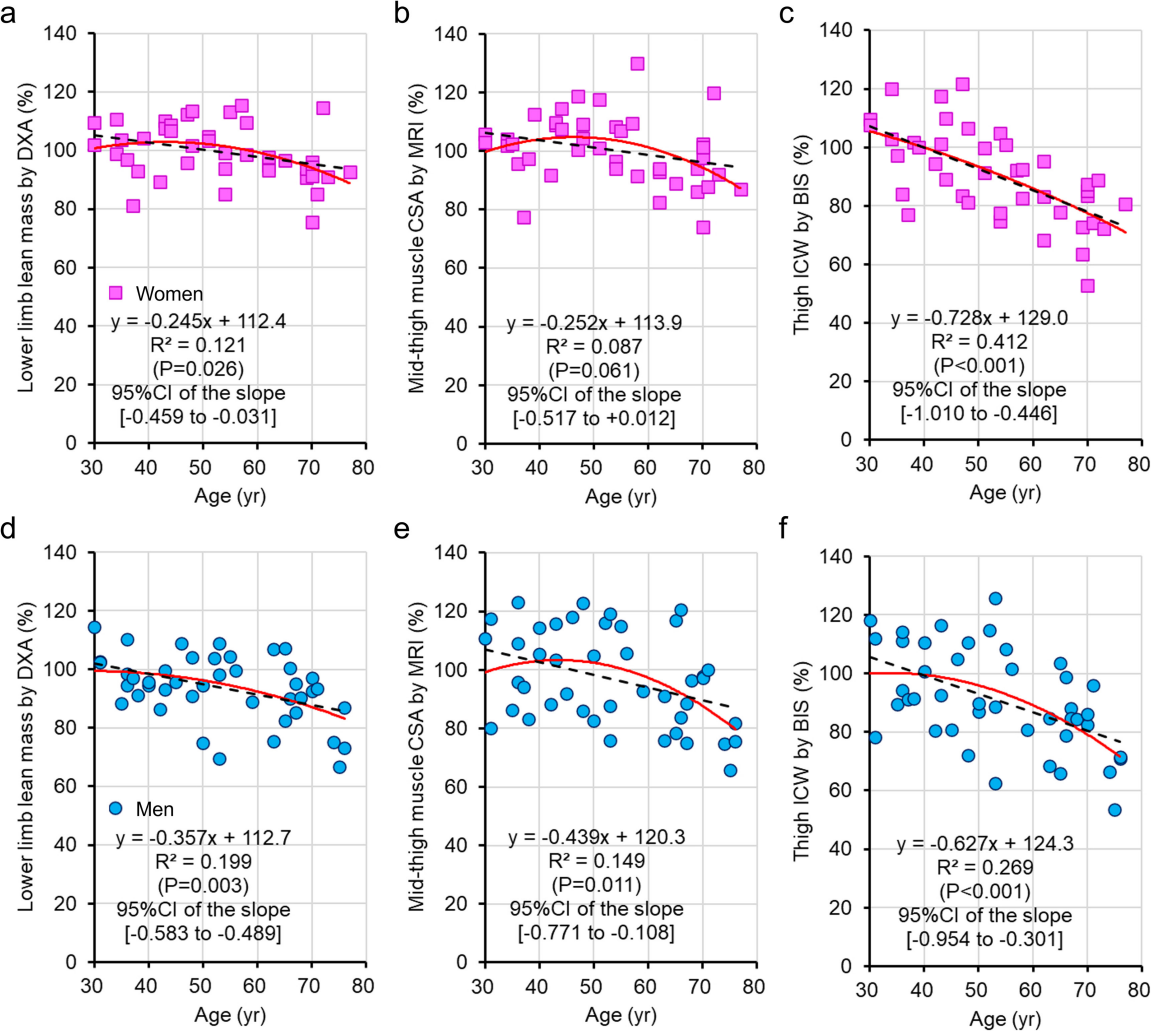
Age trends for muscle mass parameters. The age trends were determined for lower limb lean mass via dual‐energy x‐ray absorptiometry (DXA), for the cross‐sectional area (CSA) via magnetic resonance imaging (MRI) and for thigh intracellular water (ICW) via S‐BIS. The values were normalised to the relative values for each participant by using the mean score for the 30–39‐year age group for each parameter as a baseline of 100. (a–c) Women and (d–f) men.

**FIGURE 4:**
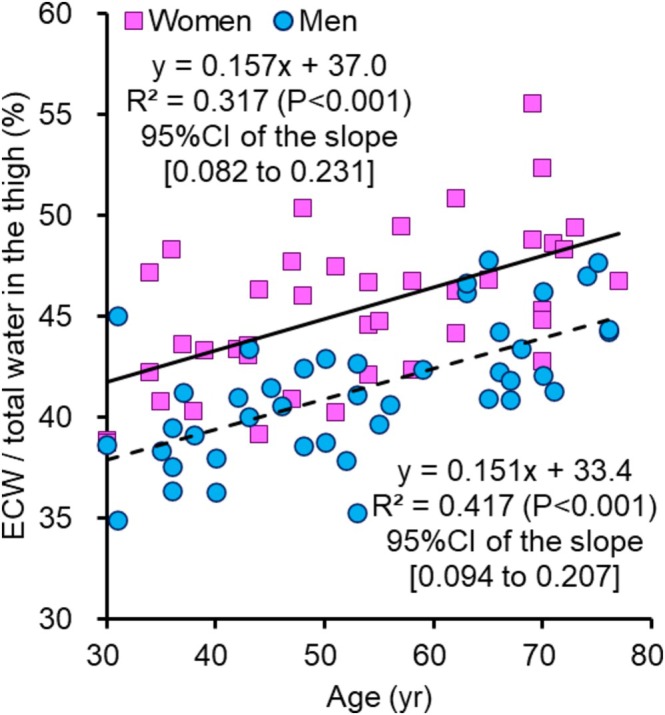
Age trends for the ratios of thigh ECW to total thigh water according to S‐BIS. The age trends of the ratios of thigh extracellular water (ECW) to thigh total water were determined by segmental bioelectrical impedance spectroscopy (S‐BIS) for women and men.

The absolute and normalised values of the lower limb lean mass obtained via DXA, the mid‐thigh CSA obtained via MRI, the thigh total water obtained via S‐BIS, the thigh ICW obtained via S‐BIS, the thigh ECW and the ECW/total water ratio in the thigh are shown in Table 2. The correlation of the absolute values of each measurement is shown in Figure 2. Figure 3 shows the age trends for the lower limb lean mass measured by DXA, the mid‐thigh muscle CSA measured by MRI and the thigh ICW measured by S‐BIS, which were normalised to the relative values for each subject by using the mean scores for the 30–39‐year age groups of the women and men, respectively, for each parameter as a baseline of 100. For women, the coefficients of determination of the regression lines for the lower limb lean mass measured by DXA, the mid‐thigh CSA measured by MRI and the thigh ICW measured by S‐BIS were 0.121, 0.087 and 0.412, respectively, with slopes of −0.245, −0.252 and −0.728, respectively (*p* values and 95% CI of the slopes are shown in Figure 3). For men, the coefficients of determination of the regression lines for the lower limb lean mass measured by DXA, the mid‐thigh CSA measured by MRI and the thigh ICW measured by S‐BIS were 0.199, 0.149 and 0.269, respectively, with slopes of −0.357, −0.439 and −0.627, respectively (*p* values and 95% CI of the slopes are shown in Figure 3). Figure 4 shows the age trends for the ratios of thigh ECW to total thigh water according to S‐BIS. The ratios of thigh ECW to total thigh water were positively correlated with age, with higher values observed at older ages for both women and men. The coefficients of determination of the regression lines for the ratios of thigh ECW to thigh total water measured by S‐BIS were 0.317 and 0.417, with slopes of 0.157 and 0.151 for women and men, respectively (*p* values and 95% CI of the slopes are shown in Figure 4).

**TABLE 2:** Muscle parameters of females and males in each age group.

Age group (years)	30–39	40–49	50–59	60–69	70–79	One‐way ANOVA (*p*)
Women						
Lower limb lean mass measured by DXA						
Absolute value (kg)	11.23 ± 0.95	11.80 ± 0.86	11.52 ± 1.02	10.61 ± 0.25	10.40 ± 1.16	0.0348
Normalised value (%)	100.00 ± 8.48	105.10 ± 7.62	102.61 ± 9.06	94.48 ± 2.26	92.59 ± 10.35	
Mid‐thigh muscle CSA measured by MRI						
Absolute value (cm^2^)	85.63 ± 7.87	91.84 ± 6.34	90.88 ± 9.84	76.82 ± 3.71	81.53 ± 10.84	0.0112
Normalised value (%)	100.00 ± 9.20	107.25 ± 7.41	106.13 ± 11.49	89.71 ± 4.33	95.21 ± 12.66	
Thigh total water measured by BIS						
Absolute value (L)	5.02 ± 0.55	5.20 ± 0.50	4.74 ± 0.46	4.30 ± 0.31	4.25 ± 0.46	0.0014
Normalised value (%)	100.00 ± 10.89	103.72 ± 9.99	94.59 ± 9.23	85.80 ± 6.19	84.76 ± 9.21	
Thigh ICW measured by BIS						
Absolute value (L)	2.88 ± 0.35	2.90 ± 0.40	2.61 ± 0.28	2.22 ± 0.30	2.25 ± 0.32	0.0004
Normalised value (%)	100.00 ± 12.27	100.57 ± 13.73	90.70 ± 9.86	76.89 ± 10.40	78.08 ± 11.01	
Thigh ECW measured by BIS						
Absolute value (L)	2.14 ± 0.28	2.31 ± 0.19	2.13 ± 0.25	2.09 ± 0.11	2.00 ± 0.18	0.1332
Normalised value (%)	100.00 ± 13.22	107.96 ± 8.91	99.84 ± 11.94	97.83 ± 5.14	93.76 ± 8.64	
ECW/total water (%)	42.58 ± 3.19	44.48 ± 3.27	44.92 ± 2.79	48.73 ± 3.69	47.29 ± 2.83	0.0097
Men						
Lower limb lean mass measured by DXA						
Absolute value (kg)	17.92 ± 1.45	17.30 ± 1.15	16.77 ± 2.29	16.59 ± 1.85	14.98 ± 1.95	0.0511
Normalised value (%)	100.00 ± 8.09	96.53 ± 6.43	93.60 ± 12.76	92.58 ± 10.31	83.60 ± 10.91	
Mid‐thigh muscle CSA measured by MRI						
Absolute value (cm^2^)	128.68 ± 18.95	135.27 ± 16.67	128.64 ± 19.16	118.23 ± 20.45	109.04 ± 16.20	0.0879
Normalised value (%)	100.00 ± 14.73	105.12 ± 12.95	99.97 ± 14.89	91.88 ± 15.89	84.73 ± 12.59	
Thigh total water measured by BIS						
Absolute value (L)	7.52 ± 0.76	7.42 ± 1.02	7.30 ± 1.18	6.87 ± 0.75	6.24 ± 0.89	0.0932
Normalised value (%)	100.00 ± 10.13	98.70 ± 13.59	97.16 ± 15.76	91.34 ± 9.95	82.98 ± 11.79	
Thigh ICW measured by BIS						
Absolute value (L)	4.60 ± 0.61	4.45 ± 0.69	4.39 ± 0.83	3.87 ± 0.54	3.46 ± 0.60	0.0147
Normalised value (%)	100.00 ± 13.17	96.63 ± 14.92	95.37 ± 18.14	84.11 ± 11.68	75.27 ± 12.98	
Thigh ECW measured by BIS						
Absolute value (L)	2.92 ± 0.23	2.97 ± 0.38	2.92 ± 0.40	3.00 ± 0.26	2.77 ± 0.32	0.7527
Normalised value (%)	100.00 ± 8.04	101.97 ± 12.98	99.99 ± 13.61	102.74 ± 8.91	95.14 ± 10.87	
ECW/total water (%)	38.96 ± 2.75	40.20 ± 2.12	40.14 ± 2.39	43.79 ± 2.42	44.70 ± 2.24	< 0.0001

*Note:* The values are expressed as the means ± standard deviations. In women, the lower limb lean mass measured via dual‐energy x‐ray absorptiometry (DXA), the cross‐sectional area (CSA) measured via magnetic resonance imaging (MRI), the total water measured via segmental bioelectrical impedance spectroscopy (S‐BIS), the thigh intracellular water (ICW) measured via S‐BIS and the ratio of ECW to total water were significantly affected by age (one‐way ANOVA, *p <* 0.05). In men, the thigh ICW measured via S‐BIS and the ratio of ECW to total water were significantly affected by age (one‐way ANOVA, *p <* 0.05).

**FIGURE 5:**
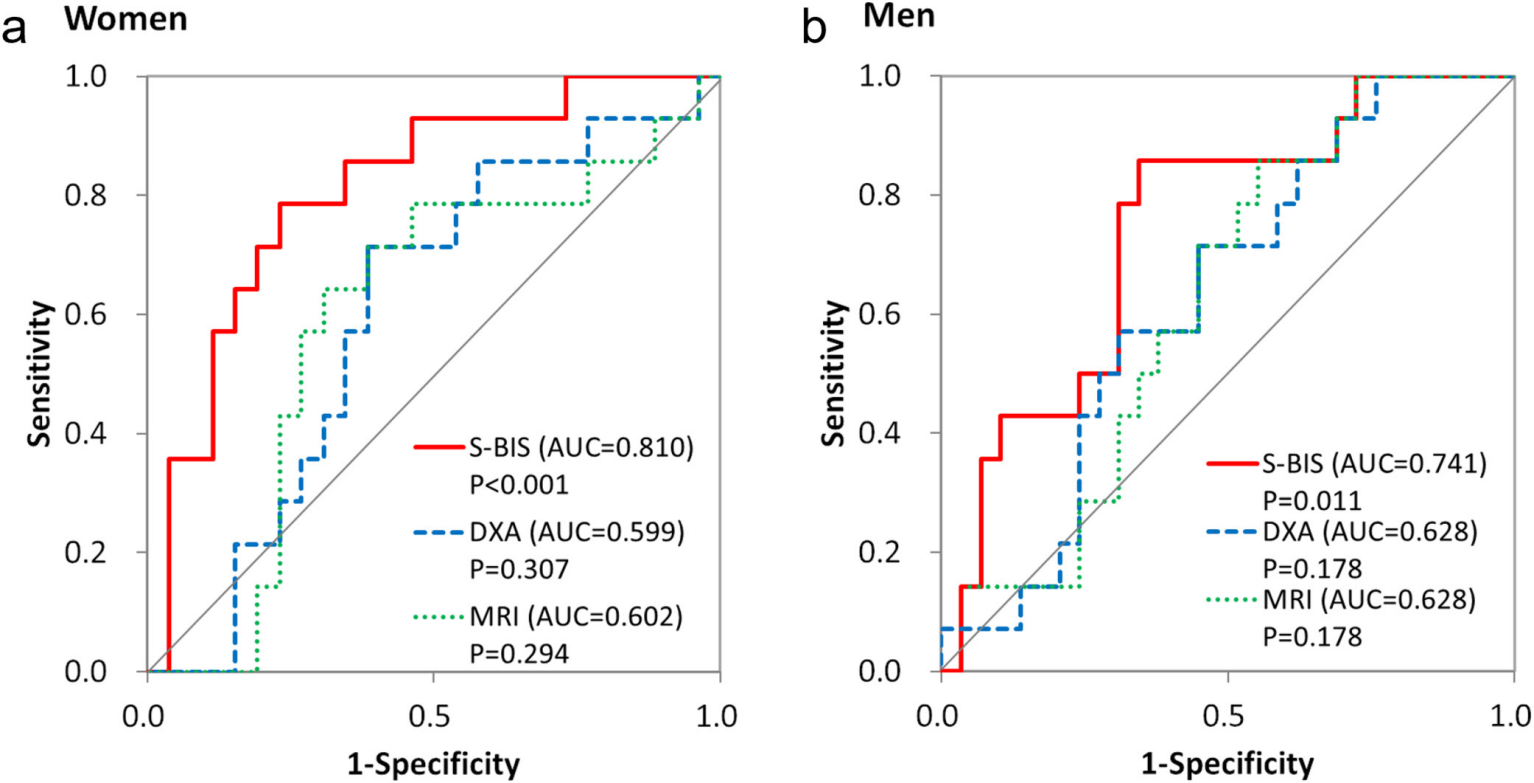
ROC curves of muscle mass parameters. The receiver operating characteristic (ROC) curves of S‐BIS, DXA and MRI with the cutoff point set at 44 years of age. (a) Women and (b) men.

**FIGURE 6:**
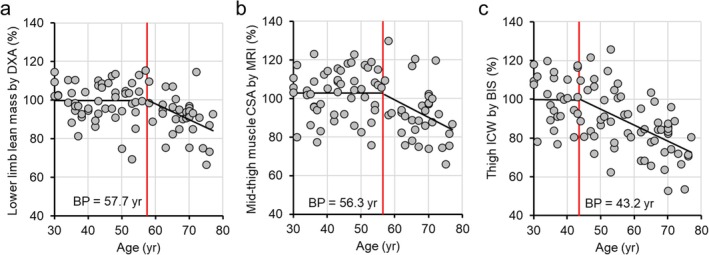
Segmented regression analysis of muscle mass parameters. Segmented regression analysis was used to determine the age breaking point for each parameter. (a) Dual‐energy x‐ray absorptiometry (DXA)–measured lower limb lean mass, (b) magnetic resonance imaging (MRI)–measured mid‐thigh cross‐sectional area (CSA) and (c) segmental bioelectrical impedance spectroscopy (S‐BIS)–measured thigh intracellular water (ICW). The values were normalised to the relative values for each participant by using the mean score for the 30–39‐year age group for each parameter as a baseline of 100. The significance level was set at *α* = 0.05.

These regression curves revealed that different parameters tended to decline at different rates with age and that there seemed to be different age points at which the parameters began to decline from their values of 100. The result of ROC analysis by setting the cutoff point at 44 years of age indicated that the AUCs of ICW measured by S‐BIS, lower limb lean mass measured by DXA and thigh CSA measured by MRI were 0.810, 0.599 and 0.602, respectively (Figure 5). In addition, we performed segmented regression analysis via SegRegA software to assess the age breaking points accurately. The age breaking points were 57.7, 56.3 and 43.2 years for the DXA‐measured lower limb lean mass, MRI‐measured mid‐thigh CSA and S‐BIS‐measured thigh ICW, respectively (Figure 6).

## 4 Discussion

The leg lean mass measured by DXA, the mid‐thigh CSA measured by MRI, the thigh TW measured by S‐BIS and the thigh ICW decreased with age in both women and men (Table 2 and Figure 3). On the other hand, the ratios of thigh ECW/TW were positively correlated with age, with higher values observed at older ages in both women and men (Table 2 and Figure 4). These results are consistent with those of previous studies [9, 13]. There were no significant differences in terms of trends between women and men. The results of the segmented regression analysis revealed that both the leg lean mass measured by DXA and the mid‐thigh CSA measured by MRI began to decrease in the late 50s (57.7 and 56.3 years old). On the other hand, the thigh ICW measured by S‐BIS began to decrease at ages as young as the early 40s (43.2 years old) (Figure 6). This finding is consistent with the results of the ROC analyses, which were conducted with 44 years as the cutoff point. The ICW measured by S‐BIS had the largest AUC and was the only parameter with *p* < 0.05 (Figure 5).

Skeletal muscle tissue is composed of myofibers, which are formed from muscle cells, and surrounding intermuscular adipose and connective tissues [7, 8]. Since skeletal muscle contractions are caused by myofibers, which are formed from muscle cells, it is believed that MCM, not total muscle mass, contributes to skeletal muscle contractions. Muscle tissue contains nearly 70% water, which is distributed intracellularly and extracellularly. It is thought that the ICW reflects the MCM [9, 10]. In this study, we measured ICW, ECW and TW separately via S‐BIS. We found that the TW and ICW contents were lower at older ages than at younger ages, and the difference was more pronounced for ICW. Since the ICW is considered to reflect contractile muscle mass, contractile muscle mass decreases at an earlier age than previously recognised by standard measurement methods, such as DXA or MRI.

As mentioned above, the traditional methods for estimating SMM (such as the measurements of lean mass via DXA and the muscle CSA via MRI, which are based on the principles of these methods) assess not only the ICW content but also the ECW content in muscle tissue [11, 12]. This may explain the delayed age‐related breaking points for DXA and MRI compared with those for S‐BIS observed in this study. For the MRI analysis, we excluded the IMAT and measured the muscle CSA. Notably, even when IMAT is excluded, MRI analysis may not accurately assess the reduction in MCM with age. This finding indicates that compositional changes other than an increase in IMAT may occur in muscle tissue. While S‐BIS has several advantages over MRI, the disadvantage of S‐BIS is that it assesses muscle mass in the entire limb and does not allow for muscle mass assessment from a single/specific muscle, which is possible with MRI. The assessment of creatine (Cr) content in human skeletal muscles is another approach for quantifying contractile muscle mass [21]. The D3‐Cr dilution method is based on the principle that approximately 95% of the total Cr pool in humans exists in muscle cells and is considered one of the methods that can precisely estimate whole‐body MCM, which, considering its measurement principle, is not affected by the ratio of the ICW to the ECW. The D3‐Cr dilution method has also demonstrated that muscle mass measurement via DXA may underestimate the effects of age‐related muscle atrophy [22]. These findings support the findings of our current study that DXA underestimates age‐related muscle atrophy because DXA is influenced not only by the ICW but also by the ECW, whereas S‐BIS can more sensitively estimate age‐related muscle atrophy by assessing the ICW and ECW separately.

Since S‐BIS measures the water content in tissues, it may be affected by the water content in fat. In the present study, intermuscular and subcutaneous fat might also have affected the measurement; however, owing to the lack of quantitative information, no correction was performed herein.

In the present study, S‐BIS findings revealed that age‐related atrophy of skeletal muscles commences in the early 40s. This finding may partially explain the previously suggested notion that the decline in skeletal muscle function starts earlier than the reduction in muscle mass [5].

The limitation of this study is that only histoanatomical evaluation was performed rather than direct assessments of muscle function or muscle strength. However, previous studies have shown a very high correlation between ICW and muscle strength [13]. In this study, the CSA measured via MRI was measured only on mid‐thigh images, whereas S‐BIS was used to measure volume. Although three‐dimensional imaging should be optimal for comparison, it is nearly impossible to apply in the clinic; therefore, two‐dimensional imaging was used in this study. Additionally, in this study, MRI measurements were performed via time‐weighted images. However, there are other methods of assessing muscle condition using MRI, such as muscle proton‐density fat fraction or diffusion tensor imaging, which may be more sensitive for detecting age‐related changes in muscle. The characteristics of each muscle mass quantification method are summarised in Table S1.

Since this study was the first to explore the age at which muscle mass starts to decline by measuring the ICW via S‐BIS, the sample size could not be calculated in advance. Furthermore, a certain number of participants were required for the analysis with SegRegA. We analysed female and male data combined to calculate the age breaking points; therefore, the breaking points could not be obtained separately for each sex. However, we do not consider this to be a significant problem in this study, as we normalised values for women and men, and the differences in absolute values were cancelled out. In addition, the age‐related changes showed similar trends for women and men. In this study, we determined the ICW decline point by using the mean scores for the 30–39‐year groups. Since the recruited subjects did not engage in regular physical exercise to exclude the effects of exercise as much as possible and to focus on changes in ageing, it is possible that the decrease in ICW could begin before the age of 30. This could suggest that the observed threshold for decline might be underestimated. Another limitation is that this study uses a cross‐sectional design; a longitudinal design would allow for a better determination of the cut‐offs for age‐related declines. Further large‐scale investigations which include muscle strength assessments, employ a longitudinal design, with a sufficient number of subjects of both sexes, such that separate analyses can be performed for women and men, including younger individuals and subjects with regular physical exercise, are needed to refine this assessment. However, we believe that this study is significant in that it conducted three simultaneous measurements to evaluate SMM in the same subjects, providing valuable insights.

In this study, we found that, compared with the lean mass measured via DXA and the muscle CSA measured via MRI, which are considered standard methods for estimating SMM, the S‐BIS method was more useful for detecting the loss of MCM, which is involved in muscle contractions. The age breaking point for the thigh ICW measured by S‐BIS showed that it began to decrease at a younger age (i.e., early 40s) than previously thought. This finding is consistent with the age at which muscle strength begins to decline, indicating that not only muscle strength but also MCM, which is associated with contractions, decreases in the 40s with ageing; however, muscle strength was not assessed in this study, and therefore, a direct comparison of age‐related declines in muscle strength and MCM was not possible, and caution should be taken when interpreting these findings. These findings suggest that it may be important to address not only the decline in muscle strength but also the decline in MCM from a relatively early age, specifically in the early 40s, to counteract the decline in quality of life among older individuals.

## Ethics Statement

The Ethics Committee of the Fukuda Clinic and Ritsumeikan University approved the study protocol in compliance with the Declaration of Helsinki. All patients provided their informed consent prior to the inclusion in the study.

## Conflicts of Interest

This work was supported by Suntory Wellness Ltd. Y.O., T.I. and T.R. are employees of Suntory Wellness Ltd., which manufactures and sells health food products. A.M. is an employee of Suntory Beverage & Food Ltd., which is the group company of Suntory Holdings Ltd., as well as Suntory Wellness Ltd. H.S. is an employee of Suntory Holdings Ltd. The other authors declare no conflicts of interest.

## Supporting information


**Table S1** **.** Characteristics of muscle mass quantification methods. MRI, magnetic resonance imaging; DXA, dual‐energy x‐ray absorptiometry; D3‐Cr dilution method, deuterium 3‐creatine dilution method; BIA, bioelectrical impedance analysis; S‐BIS, segmental bioelectrical impedance spectroscopy.
