# Supplementary material for: Early Detection of Age‐Related Decline of Muscle Cell Mass by Intracellular Water Assessment Compared With MRI or DXA
Source: J Cachexia Sarcopenia Muscle. 2025 Jun 4;16(3):e13851. doi: 10.1002/jcsm.13851 (PMC12134767; doi:10.1002/jcsm.13851)
Supplement: Supplementary file 1 — Table S1 . Characteristics of muscle mass quantification methods. MRI, magnetic resonance imaging; DXA, dual‐energy x‐ray absorptiometry; D3‐Cr dilution method, deuterium 3‐creatine dilution method; BIA, bioelectrical impedance analysis; S‐BIS, segmental bioelectrical impedance spectroscopy. [file JCSM-16-e13851-s001.docx]

**Table S1. Characteristics of muscle mass quantification methods**

|  | Measurement target | Availability at bedside or in field work | Measurement time | Invasiveness | Evaluation per site | Availability of quantification as volume | Cost |
| --- | --- | --- | --- | --- | --- | --- | --- |
| MRI | Muscle mass | Not available | Long | Not invasive | Per muscle group | Available (but need time and cost) | High |
| DXA | Lean mass | Not available | Middle | Slightly invasive | Per region | Not available | Middle |
| D3-Cr  dilution method | Muscle mass or Muscle cell mass | Available | Long | Not invasive | Whole body | Available | High |
| BIA (single-frequency) | Lean mass or  fat-free mass | Available | Short | Not invasive | Whole body or  Per region | Available | Low |
| S-BIS | Intra-cellar water or Muscle cell mass | Available | Short | Not invasive | Whole body or  Per region | Available | Low |

MRI; magnetic resonance imaging, DXA; dual-energy X-ray absorptiometry, D3-Cr dilution method; deuterium 3-creatine dilution method, BIA; bioelectrical impedance analysis, S-BIS; segmental bioelectrical impedance spectroscopy.
